# The AI Revolution in Virtual Try-Ons: A Means–End Chain Model Perspective

**DOI:** 10.3390/bs16071070

**Published:** 2026-06-30

**Authors:** Ju-Young M. Kang, Ji Young Lee, Dooyoung Choi, Sumin Helen Koo, Jeehyun Song, Youngjin Bahng

**Affiliations:** 1 Department of Family and Consumer Sciences, University of Hawai‘i at Mānoa, Honolulu, HI 96822, USA; yb7@hawaii.edu; 2 Fashion and Textile Technology Department, State University of New York (SUNY) Buffalo State, Buffalo, NY 14222, USA; leej@buffalostate.edu; 3 Department of Educational Leadership and Workforce Development, Old Dominion University, Norfolk, VA 23529, USA; dchoi@odu.edu; 4 Department of Clothing & Textiles, Yonsei University, Seoul 03722, Republic of Korea; jeehyunsong@yonsei.ac.kr

**Keywords:** artificial intelligence, clothing in relation to self, loyalty, means–end chain, quality, retail user experience, value equity, virtual try-ons

## Abstract

Leading brands have begun to implement artificial intelligence-driven virtual try-on (AI VTO) technology, which helps reduce returns and increase conversion rates, repeat purchases, and customer loyalty. This research aimed to examine how retail user experience with specific perceived quality factors of AI VTOs influences users’ value equity and downstream loyalty and to investigate the moderating effects of clothing in relation to the self as structure and concern for physical appearance, based on a *Means–End Chain* model. Data were collected from 509 U.S. online apparel shoppers using a consumer panel. Structural equation modeling and multigroup analysis were used for data analysis. This study found that pragmatic quality, hedonic quality, and customization positively affected the value equity for AI VTOs. Value equity had a positive effect on satisfaction with AI VTOs, which had a positive impact on repurchase loyalty using AI VTOs and brand loyalty. The effect of value equity for AI VTOs on satisfaction was found to be stronger among users with high levels of clothing in relation to the self as structure than among those with low levels. This study confirmed the applicability of the *Means–End Chain model* to the *quality–value–satisfaction–loyalty* chain in the AI VTO context. This study helps identify which features of AI VTO systems most significantly affect cognitive and affective assessment and behavioral outcomes.

## 1. Introduction

Artificial intelligence-driven virtual try-on (AI VTO) systems bring together computer vision, generative AI, and deep learning (Siddiquee, 2024) to generate virtual representations of products from images and videos of online consumers. The user experience of AI VTO systems begins with the system capturing an image of the user or with the user uploading their chosen image or video (GlamAR, 2024). Computer vision algorithms analyze user images to ascertain body dimensions, clothing attributes, facial features/contours, skin tones, and other attributes (Sisohor et al., 2025). AI-driven models provide realistic selected product views by modifying body and product size, fit, color, and placement (Rincon, 2025). Subsequently, machine learning algorithms optimize the experience of personalization and the results based on user engagement, interactions, preferences, and opinions (GlamAR, 2024; Rincon, 2025).

Well-known leading brands (e.g., Sephora, Warby Parker, L’Oréal, IKEA, Nike) have begun implementing AI-driven virtual try-on (AI VTO) technology. Google also provides virtual try-ons from brands (e.g., Anthropologie, American Eagle Outfitters, Everlane, H&M, LOFT) that integrate advanced generative AI models via Google Search and the Google Shopping tab. When users choose clothing items marked with a “try it on” symbol in the Google Shopping tab, they can view clothing on the AI model that matches their personal body types and preferences (Rincon, 2025). Furthermore, when users of an AI fashion app Doji upload their selfies and full-body photos to the app, they can view virtual model avatars via this app (Bradley & Stokes, 2025). In addition, Amazon recently introduced an AI fitting tool that unifies sizing (Chaudhri, 2024). Thus, AI VTO technology entices users with realistic try-ons of fashion and cosmetics and offers a novel, personalized, and engaging shopping experience. This not only enhances customer engagement and loyalty but also achieves marketing goals, such as higher conversion rates and increased repeat purchases (Siddiquee, 2024).

Along with the rapid growth of virtual try-on systems in e-commerce, researchers are giving increasing attention to this area. In the conventional VTO context without AI, earlier work has shown that offering VTO significantly increases consumers’ curiosity about products and their purchase intentions (Beck & Crié, 2018), reduces product return rates, and enhances customer satisfaction (S. Yang & Xiong, 2019). Previous studies demonstrated the effects of ease of use (Mollel & Chen, 2025), usefulness (Sekri et al., 2025), informativeness (Micheletto et al., 2025), perceived entertainment (J. Kim & Forsythe, 2009), and playfulness (Q. Jiang et al., 2022) of using VTOs on behavioral outcomes. While some studies reported positive effects on enjoyment when using VTOs (Nawaz et al., 2025), others found no significant impact (Sekri et al., 2025). In addition, recent studies have examined the integration of VTO into eyewear e-commerce platforms (Abisheak et al., 2023), the effect of technology readiness on consumer acceptance of 3D virtual fitting technologies in cross-border online shopping (H. Yang & Kim, 2024), and the effect of self-presence facilitated by augmented reality VTO on self-explorative engagement and brand attitudes (Lavoye et al., 2023). However, the existing literature remains limited by examining only selective features of VTO without AI rather than a comprehensive set of attributes integrated with AI. Recent empirical studies have not yet investigated the potential role of the AI-driven functionalities of VTO systems in building ongoing consumer behaviors such as repurchasing and loyalty. Relatively few studies have empirically identified which features of AI-driven VTOs most significantly affect cognitive and affective assessment and behavioral outcomes. To address these gaps, this study aims to understand how consumers perceive the quality of AI-driven VTO as a retail user’s experience and how various AI VTO features contribute to value, satisfaction, and loyalty of retail users.

Customer loyalty is defined as the long-term behavior of repeat patronage, shaped by a customer’s attitude toward the product or service (Bloemer & Kasper, 1995; Dick & Basu, 1994). Understanding and identifying how AI VTO features translate into users’ loyalty based on the *quality–value–satisfaction–loyalty* chain as the retail user experience model is critical because this chain validates investment in retail user experience design and AI capabilities as a driver of loyalty. Users’ *loyalty* demonstrates their psychological commitment to AI VTO and the effectiveness of user experience design. It enables service providers and marketers to target and retain high-potential user segments and ultimately results in a high return on investment. Furthermore, understanding the moderating effect of identity and appearance-related motivations, such as *clothing in relation* to self and concerns for physical appearance, is critical because these factors demonstrate consumer segments prone to higher perceived value, satisfaction, and loyalty. These insights enable retailers to easily develop strategic design, marketing, and communication strategies. This led to the development of the following research questions for the retail user experience model:

**RQ1:**  *How does retail user experience with perceived quality of AI VTOs affect their value equity?*

**RQ2:**  *Does this value equity influence user satisfaction with AI VTOs, ultimately resulting in increased loyalty if there is an impact?*

**RQ3:**  *Do clothing in relation to self and concern for physical appearance moderate the relationship between their value equity and satisfaction with AI VTOs?*

Accordingly, the research objectives were to examine (1) whether retail user experience with perceived quality of AI VTOs, such as pragmatic quality (H1), hedonic quality (H2), aesthetic quality (H3), interactivity (H4), and customization (H5), was related to value equity for AI VTO (H1–H5); (2) whether value equity was related to satisfaction with AI VTO (H6); (3) whether satisfaction was related to repurchase loyalty using AI VTO and brand loyalty (H7); and (4) the moderating effects of clothing in relation to self as structure and concern for physical appearance (H8-H9). The findings of this study contribute to the further theoretical understanding of AI VTOs. First, this study helps identify which system features most significantly affect cognitive and affective assessment and behavioral outcomes by empirically testing how specific perceived quality factors of AI virtual try-ons (VTOs) influence users’ value equity and downstream loyalty. This insight facilitates the development of more effective retail experiences and promotional strategies. Second, this research establishes a stronger psychological foundation by examining the moderating factors, both clothing in relation to self and concern for physical appearance. These elements demonstrate how personal identity and physical appearance-related motivations form the relationship between customers’ perceived value and satisfaction. This contribution enables more targeted segmentation and personalization strategies in AI VTO development. Third, our implications can be applied in practice to develop effective strategies for quickly adapting to changing customer needs and establishing a strong market presence in AI VTOs.

## 2. Conceptual Background

This study applied a *Means–End Chain* model (Gutman, 1982) to the *quality–value–satisfaction* framework (Wang et al., 2004; Zeithaml, 1988) and the *quality–value–satisfaction–loyalty* chain (Gallarza et al., 2013) in the context of AI VTOs. Specifically, the *Means–Ends Chain* theory explains how and why consumers choose products or services by linking product attributes to personal values. The model posits that consumer goals determine their purchasing decisions. It explains that consumer behavior is goal-oriented, with goals determining purchasing decisions. These goals encompass obtaining a functional advantage and aligning purchase decisions with personal values. The model views the attributes of a product (means) as enabling the fulfillment of customer values (desirable end-states) (Gutman, 1982); in other words, product attributes serve as the means through which consumers achieve their valued ends. Thus, consumer values greatly influence decision-making patterns (Gutman, 1982). Prior researchers utilized the Means–Ends Chain framework within e-commerce contexts to investigate the influence of product or platform characteristics on consumer values in multi-channel shopping (Hsiao et al., 2012), online group purchasing (Xiao et al., 2017), electronic word-of-mouth intentions (Phan et al., 2019), cross-border e-commerce (Xu et al., 2021), and augmented reality marketing (Kumar et al., 2024). In this study, retail user experience with perceived quality of AI VTOs was examined as a means state. Value equity for AI VTO was investigated as achieving the desired end state. While making a purchasing decision, consumers consider how the retail user experience with perceived quality of AI VTOs would result in specific benefits that would eventually help them enhance their value equity, satisfaction, repurchase loyalty, and brand loyalty. In our proposed model, retail user experience was operationalized as five exogenous constructs as means state: (1) pragmatic quality, (2) hedonic quality, (3) aesthetic quality, (4) interactivity, and (5) customization of AI VTOs. Value equity for AI VTO was examined as achieving the desired end state.

Furthermore, this study employed the *quality–value–satisfaction* framework (Wang et al., 2004; Zeithaml, 1988) and the *quality–value–satisfaction–loyalty* chain (Gallarza et al., 2013) to develop the proposed model. Extending the *quality–value–satisfaction* framework (Wang et al., 2004; Zeithaml, 1988), the *quality–value–satisfaction–loyalty* chain (Gallarza et al., 2013) posits that higher perceived quality enhances perceived value (i.e., consumers’ trade-off between benefits and costs), which boosts satisfaction, and satisfied customers are more likely to show loyal behaviors (Gallarza et al., 2013). Previous researchers used the *quality–value–satisfaction* framework (Wang et al., 2004) and the *quality–value–loyalty* framework (Fuentes-Blasco et al., 2010; Hanaysha et al., 2025; L. Jiang et al., 2016) in the context of e-commerce. The *service quality–satisfaction–loyalty* framework has also been applied to global e-commerce sites (Manisa & Sari, 2023), liner shipping e-commerce platforms (Chao et al., 2024), and cross-border e-commerce (Do et al., 2023). In AI VTO settings, a higher perceived quality may lead to a higher perceived value equity, which in turn may lead to greater satisfaction, ultimately resulting in loyalty. In our proposed model, higher perceived quality may lead to higher perceived value equity, which in turn may lead to greater satisfaction, ultimately resulting in loyalty in AI VTO settings.

## 3. Hypothesis Development

### 3.1. Pragmatic Quality of AI Virtual Try-Ons

Pragmatic quality comprises the clear, useful, and controllable features of a product that meet users’ behavioral goals in terms of effectiveness, efficiency, and satisfaction (Hassenzahl, 2009, 2003). In this study, pragmatic quality of AI VTO refers to how practical the system is perceived to be, as well as its reliability, usefulness, informativeness, and practicality, thereby enabling users to make clear decisions based on engagement with the virtual try-on experience. Value equity indicates how well the customer evaluates the price of an item as it compares to its value. A high-value equity evaluation has a high price/quality ratio (Vogel et al., 2008). Rosenbaum and Wong (2010) define value equity as a combination of the consumer’s perception of the quality and convenience of the service, as well as price. Value equity is represented as the consumer’s objective assessment of how useful a virtual try-on service is when considering quality and benefits versus price. Previous researchers demonstrated that pragmatic quality drives positive user experience and brand outcomes. In the augmented reality context, the pragmatic quality of augmented reality has an impact on the user’s positive experience (Kazmi et al., 2021). Lavoye et al. (2023) found that pragmatic realism, like the experience of self-presence during consumers’ use of VTOs in AR environments, has a positive effect on self-explorative engagement, which improves brand attitude via brand cognitive processing. In VTO and virtual fitting technology contexts, pragmatic attributes like perceived usefulness (Ivanov et al., 2023; H. Yang & Kim, 2024) and perceived informativeness (Chen et al., 2024) have a strong positive influence on attitude, consumers’ purchasing decisions, and adoption intentions. In the AI-powered try-on technology context, Gao and Liang (2025) found that the perceived utilitarian value of AI-powered try-on technology indirectly affects online consumers’ impulsive buying intention via enhanced utilitarian and hedonic value. It is presumed that consumers who see the pragmatic quality of the AI VTO experience will value its qualities, such as being worthwhile, practical, and appealing; therefore, the following hypothesis was formulated:

**H1.**  *The pragmatic quality of AI virtual try-ons has a positive influence on value equity for AI virtual try-ons.*

### 3.2. Hedonic Quality of AI Virtual Try-Ons

User experience involves “emotional reactions and benefits” (Hassenzahl & Tractinsky, 2006; Poushneh & Vasquez-Parraga, 2017, p. 230). User experience with AI VTO can be enriched by improving hedonic quality through emotional stimulation, rational stimulation, and identification with the system. Hedonic quality, influenced by emotional stimulation and rational stimulation, pertains to fulfilling human needs for novelty and challenge, while identification focuses on fulfilling human needs as self- expression (Hassenzahl, 2003). In the context of augmented reality, Poushneh and Vasquez-Parraga (2017) identified that consumers’ perceived hedonic quality (through identification and stimulation) leads to their satisfaction and willingness to purchase. In the VTO context, perceived enjoyment (i.e., the degree to which an individual perceives an activity as enjoyable and fulfilling) of online shopping using 3D virtual fitting technologies (Chen et al., 2024; H. Yang & Kim, 2024), curiosity, novelty, and emotional value (Chen et al., 2024) are the determinants of attitude, VTO adoption intentions, and purchase decisions. Therefore, it is expected that consumers who see the hedonic quality of the AI VTO experience will value its qualities, such as being worthwhile, reasonable, and attractive. The following hypothesis was formulated:

**H2.**  *The hedonic quality of AI virtual try-ons has a positive influence on value equity for AI virtual try-ons.*

### 3.3. Aesthetic Quality of AI Virtual Try-Ons

The aesthetic quality of user experience involves “pleasurable experiences” (Poushneh & Vasquez-Parraga, 2017, p. 230). In this study, the aesthetic quality of the retail user experience with AI VTOs consists of aesthetic quality by affect (i.e., emotional state or tendency) and aesthetic quality by cognition (i.e., general mental processing). In the augmented reality virtual try-on context, previous studies demonstrated that the aesthetic quality of augmented reality has a positive and significant association with user satisfaction and user willingness to buy (Poushneh & Vasquez-Parraga, 2017), attitudes (Q. Jiang et al., 2022), and positive experience (Kazmi et al., 2021). In the VTO context, Chen et al. (2024) documented that perceived pleasure, vividness (i.e., “the graphical effects in terms of realism of 3D images and visual appeal of the graphical look,” [p. 9]), and perceived playfulness (i.e., “the extent to which an individual perceives an activity as enjoyable, engaging, and fun,” [p. 9]) are the key determinants influencing consumers’ purchasing decisions and virtual try-on adoption intentions. Thus, it is anticipated that consumers who notice the aesthetic quality of AI VTOs will perceive them as having higher value and as being more worthwhile, practical, and engaging. The following hypothesis was developed:

**H3.**  *The aesthetic quality of AI virtual try-ons has a positive influence on value equity for AI virtual try-ons.*

### 3.4. Interactivity of AI Virtual Try-Ons

Perceived interactivity denotes the user’s perception of bidirectional, controlled, and responsive communication within computer-mediated settings; it constitutes a psychological state encountered during media interaction and represents an experiential phenomenon (Chen et al., 2024; Mollen & Wilson, 2009; Wu, 2006). Previous studies demonstrated that enhanced image interactivity technology increases the novelty and entertainment value of shopping (Li et al., 2011), enhances shopping enjoyment and reduces perceived risk, which in turn positively influences attitudes toward the online retailer (H. H. Lee et al., 2010) and increases value equity in online social co-creation contexts (Kang, 2014). In AR-enabled shopping contexts, consumers’ perceived interactivity indirectly and directly enhances their attitudes (H. Lee et al., 2021), choice confidence and purchase intention (Kowalczuk et al., 2021), and intrinsic value and attitude (Q. Jiang et al., 2022). In the VTO context, interactivity is one of the determinants affecting consumers’ purchasing decisions and intention to adopt VTOs (Chen et al., 2024). Gao and Liang (2025) found that interactive control of AI-powered try-on technology indirectly affects online consumers’ impulsive buying intention via enhanced utilitarian and hedonic value. Thus, it is anticipated that consumers who recognize the interactivity of AI VTOs will perceive it as highly valuable, worthwhile, reasonable, and attractive. The following hypothesis was formulated:

**H4.**  *Interactivity of AI virtual try-ons has a positive influence on value equity for AI virtual try-ons.*

### 3.5. Customization of AI Virtual Try-Ons

Customization is a marketing approach that enables customers to personalize products or services (Liao et al., 2019). Avatar identification is the phenomenon that occurs when consumers perceive an avatar as a representation of themselves and an extension of their identity (Teng, 2019). In the context of augmented reality-based shopping, perceived personalization and customization features indirectly affect a favorable attitude (Q. Jiang et al., 2022) and telepresence and product liking, which in turn enhance choice confidence and repurchase and reuse intentions (Kowalczuk et al., 2021). In the virtual reality and VTO apps contexts, customization features (i.e., avatar personalization and avatar identification) satisfy consumers’ intrinsic needs, which in turn affect intention to use (Lau & Ki, 2021) and enhance consumer inspiration, which in turn results in adoption intention (Tawira & Ivanov, 2023). Gao and Liang (2025) documented that personalized AI-powered try-on technology generates virtual representations of users, provides customized outfit recommendations, and enhances satisfaction with the fit as well as a sense of identity and exclusivity. Thus, it is expected that consumers who identify customization of AI VTOs will perceive it as highly valuable, worthwhile, reasonable, and attractive. Thus, the following hypothesis was formulated:

**H5.**  *Customization of AI virtual try-ons has a positive influence on value equity for AI virtual try-ons.*

### 3.6. Value Equity for AI Virtual Try-Ons

Value equity is considered a reliable measure of a firm’s capability to fulfill customer expectations regarding its products and services, which results in influencing future sales (Vogel et al., 2008). Previous researchers found that value equity has a positive influence on a customer’s switching propensity and loyalty intentions (Rust et al., 2001, 2004), which in turn results in future sales (Vogel et al., 2008) and commitment and repurchase loyalty in social co-creation platforms (Kang, 2014). Consumers’ utilitarian and hedonic values have an impact on behavioral intention toward AR-based mobile shopping (Yoo, 2023). Based on these research findings, it was anticipated that the perception of a high level of value equity of AI VTOs would lead to a high level of satisfaction with AI VTOs. Therefore, the following hypothesis was developed:

**H6.**  *Value equity for AI virtual try-ons has a positive influence on satisfaction with AI virtual try-ons.*

### 3.7. Satisfaction with AI Virtual Try-Ons, Repurchase Loyalty, and Brand Loyalty

Satisfaction refers to the customer’s positive or negative emotional experience when they evaluate how well a product or service actually performs compared to how well they expected it to perform (Chen et al., 2024). In this study, user satisfaction is how well users rate their experience with purchase and consumption using AI VTOs. Previous studies demonstrated that customer satisfaction has an impact on repurchase loyalty within a relative attitudinal framework (Olsen, 2002), patronage behavior and retailer loyalty (M. J. Kim & Mullis, 2010), and consumers’ purchasing decisions and virtual try-on adoption intentions (Chen et al., 2024). In the context of AR-based virtual fitting rooms and virtual try-ons, consumers’ attitude (i.e., utilitarian and hedonic components) has a positive influence on their intention to adopt (H. H. Lee et al., 2010) and intention to continue using (Q. Jiang et al., 2022). Lau and Ki (2021) found that the fulfillment of consumers’ intrinsic needs through a virtual reality (VR) fashion app has a positive impact on their intention to use a VR fashion app. Based on these research findings, it is anticipated that a high level of satisfaction with AI VTOs will lead to high levels of repurchase loyalty using AI virtual try-ons and brand loyalty. Therefore, the following hypothesis was developed:

**H7.**  *Satisfaction with AI virtual try-ons positively influences (a) repurchase loyalty using AI virtual try-ons and (b) brand loyalty.*

### 3.8. Clothing in Relation to the Self as a Structure

Clothing in relation to the self as a structure is one of the six dimensions of proximity of clothing to the self (Sontag & Lee, 2004). It is defined as “the psychological closeness of clothing to self and indicated by the extent to which clothing is perceived as one with the self or as a component of the self” (Sontag & Schlater, 1982, p. 1). Clothing validates oneself through communication, perception, and judgment, allowing for experimentation with different selves and role-taking, which involves self-communication and responses to others’ judgments (Sontag & Lee, 2004). Although existing research related to the *proximity of clothing to the self* did not focus on e-commerce settings, some evidence related to clothing and self-alignment exists, such as fashion consciousness, individualism, and self-congruence. Specifically, Chen et al. (2024) documented that fashion consciousness (i.e., assessing individuals’ ideas and attitudes toward fashion to influence individual decision-making) and individualism are the key determinants influencing consumers’ purchasing decisions and virtual try-on adoption intentions. Ha and Im (2012) demonstrated that self-congruence moderates the relationship between utilitarian shopping value and satisfaction. M. J. Kim and Mullis (2010) found that self-congruity (i.e., the consumer’s self-image and the perceived retail patron image) has a positive influence on satisfaction with the retailer and retailer loyalty. Thus, clothing and self-alignment enhance value outcomes and positively impact shopping value, satisfaction, and loyalty intentions. It is expected that the relationship between value equity and satisfaction will be stronger for users with a high level of clothing in relation to the self as a structure, and it will be weaker when users have a low level of clothing in relation to the self as a structure. The following hypothesis was formulated:

**H8.**  *Clothing in relation to the self as a structure moderates the positive relationship between value equity for and satisfaction with AI VTOs.*

### 3.9. Concern for Physical Appearance

Concern for physical appearance is one of the four dimensions of vanity (Durvasula & Lysonski, 2008). Previous studies demonstrated the effects of concerns about garment fit and size, body satisfaction/esteem, and fit confidence on behavioral outcomes, such as enjoyment, attitude, adoption, and usage intention toward virtual try-on technology. Shin and Baytar (2014) found that individuals with a higher degree of concern about garment fit and size are more willing to use a virtual try-on model. Individuals with higher body satisfaction tend to perceive more enjoyment from the virtual product experience in online apparel shopping, which helps them to develop more positive attitudes toward products and increase their online purchase intention (Yu & Damhorst, 2015). S. Yang et al. (2023) identified that virtual fitting rooms negatively affect users with higher body mass indices (BMIs) and that BMI moderates the effectiveness of virtual fitting rooms. Chen et al. (2024) documented that body esteem or body satisfaction is found to be a determinant of consumers’ adoption intentions for VTOs and their purchase decisions. Furthermore, fit confidence and body esteem have a positive impact on attitude toward AR-based VTO and purchase intention (Mollel & Chen, 2025). Thus, it is expected that the relationship between value equity and satisfaction with AI VTOs will be stronger for users with a low level of concern for physical appearance and weaker for those with a high level of concern. The following hypothesis was developed:

**H9.**  *Concern for physical appearance moderates the positive relationship between value equity for and satisfaction with AI VTOs.*

## 4. Methodology

### 4.1. Sample and Data Collection

**Figure 1:**
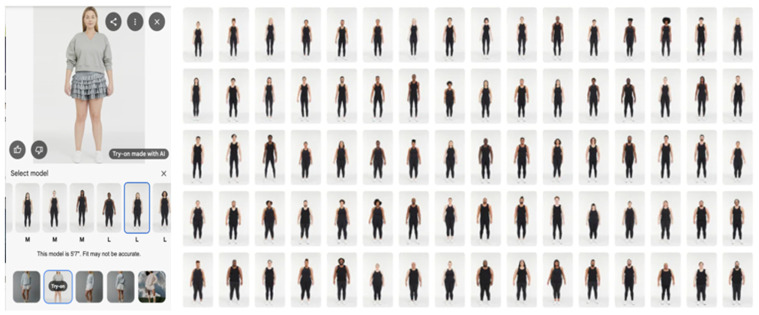
AI-driven virtual try-ons and generative AI models.

Data were collected from U.S. online apparel shoppers (*n* = 509) using a consumer panel via an online self-administered survey. Non-probability, recruitment-based, opt-in, and quota-based convenience samplings were used via a pre-existing consumer panel managed by a market research company. A market research company recruited consumer panel members from diverse states in the U.S. through email invitations. Our participants were part of a pre-existing consumer panel, had signed up to take surveys, and responded to this email invitation. Email invitations were distributed via the market research company’s email software tool to a pool of eligible panelists who met the study’s initial criteria. These email invitations contained the qualifications for participation as a purposive screening process and a link to the online questionnaire. Also, this market research company used quotas (i.e., gender, age, and ethnicity) to match the sample to the U.S. consumer population distribution and ultimately reduce bias resulting from convenience sampling. No post-stratification weighting was applied. The informed consent form was displayed on the first page of the survey. Our participants viewed a video showing how to use the AI VTO feature offered by the fashion brand on Google. They engaged with an AI VTO that Google provides for at least 3 min. This setting resembled actual consumer behavior in the AI VTO context (see Figure 1). Then, our participants answered a yes/no question to confirm that they had interacted with the AI VTO for the required three minutes. Participants who successfully finished this three-minute task were then prompted to complete an online self-administered questionnaire.

### 4.2. Measurement

Reliable measurement items were selected based on a review of the literature and were modified to fit the current AI VTO context. To ensure the adapted measurement items properly address the AI VTO context, four scholars/experts reviewed the measurement items, and a pilot test with 20 undergraduate students was conducted as content and face validation. Measures for (1) pragmatic quality (18 items, four dimensions: practicality, reliability, information, and usefulness), (2) hedonic quality (15 items, three dimensions: identification, emotional stimulation, and rational stimulation), and (3) aesthetic quality (7 items, two dimensions: cognition and affect) were adapted from Hassenzahl (2003) and Poushneh and Vasquez-Parraga (2017) to fit the AI VTO context. Sample items were “Using AI VTO is practical” for pragmatic quality, “Using AI VTO is innovative” for hedonic quality, and “Using AI VTO is aesthetically pleasing” for aesthetic quality. These were measured using a bipolar semantic differential 7-point scale method.

In addition, our participants responded to the following items using a 5-point Likert scale (1 = strongly disagree, 5 = strongly agree): Measures for (4) perceived *interactivity* (four items) and (5) customization (four items) were adapted from Pillai et al. (2020) to fit the AI VTO context. An exemplary item of interactivity was “Using AI VTO is very engaging,” and an exemplary item of customization was “Using AI VTO is perfectly suitable for my shopping requirements.” Measurement items for (6) value equity (four items) were adapted from Vogel et al. (2008) to fit the AI VTO context. An exemplary item was “How would you rate your overall shopping experience through the AI VTO? (Extremely poor value to Extremely good value).” Measurement items for (7) satisfaction (three items) originated from Poushneh and Vasquez-Parraga (2017) and Collier and Bienstock (2006). An exemplary item was “In general, I am satisfied with this AI-powered virtual try-on.” Measurement items for (8) repurchase loyalty (four items) were adapted from Chaudhuri and Ligas (2009), Kang (2014), and Olsen (2002). A sample item was “I will use AI-powered virtual try-ons the next time I buy apparel.” A measure for (9) brand loyalty (three items) originated from Eastlick and Feinberg (1999). An exemplary item was “I consider myself to be loyal to this brand’s offline and online stores.” Measurement items for (10) clothing in relation to the self as structure (six items, one of the subscales of proximity of clothing to self) originated from Sontag and Lee (2004). A sample item was “What I wear is consistent with who I am.” Measurement items for (11) concern for physical appearance (five items, one of the subscales of vanity) originated from Durvasula and Lysonski (2008). An exemplary item was “Looking my best is worth the effort” (see Table 1).

## 5. Results

### 5.1. Participant Characteristics

The total of 509 participants comprised 47.5% female and 52.5% male individuals. The age of our participants ranged from 18 to 54 years. Regarding ethnicity, 61.9% were Caucasian, followed by African American (15.7%) and Asian/Pacific Islander (8.8%). Our participants held a high school education (38.5%) or a bachelor’s degree (29.7%) as their highest completed education. A range of personal income levels was represented, with 11.2% of participants having incomes between $20,000 and $29,999 and under $10,000, followed by incomes between $30,000 and $39,999 (10.8%) and incomes between $50,000 and $59,999 (9.6%).

### 5.2. Exploratory Factor Analysis

Exploratory factor analysis (EFA) with varimax rotation was conducted to ensure that the items comprising a scale measured only one dimension or concept at a time (i.e., unidimensionality within each construct). Any factor loading greater than 0.40 was assumed to have practical significance (Hair et al., 2010). Due to the low factor loadings and cross-loadings, one item of the *pragmatic quality* measure was dropped. At this stage, all factors explained 81.21% of the total variance in the data, with factor loadings ranging from 0.49 to 0.79, and had an eigenvalue greater than one. Table 1 exhibits the results of the EFA.

### 5.3. Validity and Reliability of the Measurement

The results of confirmatory factor analysis indicated that the measurement model had a good fit (*χ*^2^ = 3545.56 with 1767 *df*, *χ*^2^/*df* =2.01, CFI = 0.96, NNFI = 0.95, IFI = 0.96, RMSEA = 0.045, and SRMR = 0.020). The reliability and convergent validity of the constructs were assessed by composite/construct reliability and average variance extracted (AVE). All items loaded on the intended constructs significantly at the *p*-value < 0.001, and the standardized factor loadings of the items ranged from 0.65 to 0.83. The recommended composite/construct reliability value for each construct is suggested to be above 0.70, and the AVE value for each construct is above 0.50 (Hair et al., 2010). In this study, the composite reliability of each construct was assessed and ranged from 0.80 to 0.96. The AVE ranged from 0.58 to 0.66 (see Table 1). All AVEs for the constructs were greater than their squared correlations, which proved discriminant validity among constructs (Fornell & Larcker, 1981) (see Table 2).

### 5.4. Common Method Bias

As specified by Harman’s one-factor test, if a common method bias exists, a single factor will account for more than 50% of the variance in a principal component factor analysis with no rotation (Harman, 1976). In this study, the largest factor contributed to 43.17% of the total variance. This study identified that no single factor explained greater than 50% of the variance, and it suggested that common method bias was unlikely to be present in this study.

### 5.5. Hypothesis and Model Testing

**Figure 2:**
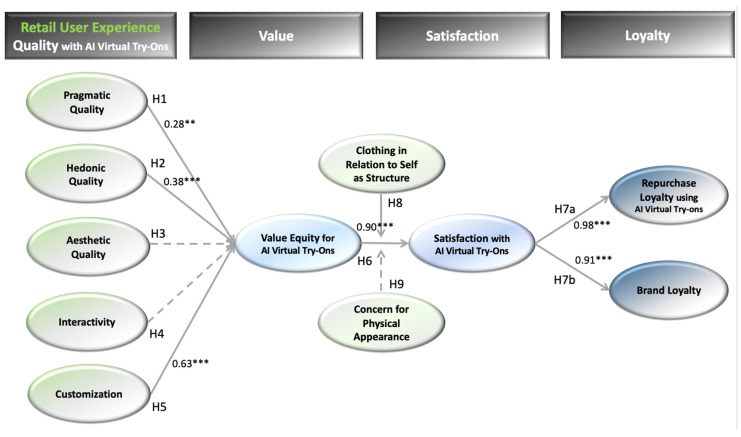
*Final Model.* All are standardized estimates. Bold paths indicate significance. ** *p* < 0.01, *** *p* < 0.001.

The proposed model was tested using structural equation modeling with maximum likelihood estimation (see Figure 2 and Table 3). The structural model demonstrated a good fit with the data (*χ*^2^ = 3875.04, *df* = 1785, *χ*^2^/*df* = 2.17, CFI = 0.95, NNFI = 0.95, IFI = 0.95, RMSEA = 0.048, and SRMR = 0.034). For H1–H5, *pragmatic quality* (*β* = 0.28, *t* = 2.90, *p* < 0.01; H1 supported), *hedonic quality* (*β* = 0.38, *t* = 4.73, *p* < 0.001; H2 supported), and *customization* (*β* = 0.63, *t* = 3.93, *p* < 0.001; H5 supported) had a positive impact on *value equity* for AI VTOs. However, aesthetic quality (H3) and interactivity (H4) showed no statistically significant impacts. For H6, *value equity* positively affected *satisfaction* with AI VTOs (*β* = 0.90, *t* = 25.64, *p* < 0.001; H6 supported). For H7, *satisfaction* had a positive impact on *repurchase loyalty* using AI VTOs (*β* = 0.98, *t* = 27.82, *p* < 0.001; H7a supported) and *brand loyalty* (*β* = 0.91, *t* = 23.53, *p* < 0.001; H7b supported).

To test the moderating effect, SEM multigroup analysis was conducted. The hypothesis regarding the moderating effects of *clothing in relation to self as structure* on the relationship between *value equity and satisfaction* was supported (H8). The model fit difference from the comparison of the two groups (low versus high; median split = 3.83) indicated that the coefficients for the two groups were significantly different for the relationship between *value equity and satisfaction* (Δ*χ*^2^ = 4.98, Δ*df* = 1, *p* = 0.026). The estimated coefficient of the effect of *value equity* on satisfaction increased from 0.52 (low) to 0.61 (high). In other words, the effect of *value equity* on satisfaction is likely to be more evident (or stronger) for those with high levels of *clothing in relation to self as structure* than those with low levels of *clothing in relation to self as structure* (see Table 4). Furthermore, two-way ANOVA is one of the statistical tests for testing moderating effects (J. S. Kim et al., 2001). To examine the moderating effect of *clothing in relation to self as structure*, a two-way ANOVA was performed as well. *Value equity* (scale/continuous variables) was dichotomized using a median split at 4.75. One moderator, *clothing in relation to self as structure,* was dichotomized using a median split at 3.83. There was not a significant interaction between *clothing in relation to self as structure* and *value equity* on *satisfaction*, *F* (1, 505) = 10.59, *p* = 0.059. *Clothing in relation to self as structure* did not moderate the link between *value equity* and *satisfaction*.

However, the hypothesis regarding the moderating effects of *concern for physical appearance* on the relationship between *value equity and satisfaction* was not supported (H9). The model fit difference from the comparison of the two groups (low versus high; median split = 3.6) indicated that the coefficients for the two groups were not significantly different for the relationship between *value equity* and *satisfaction* (Δ*χ*^2^ = 2.28, Δ*df* = 1, *p* = 0.131). Furthermore, a two-way ANOVA was performed. Value equity (scale/continuous variables) was dichotomized using a median split at 4.75. The other moderator, *concern for physical appearance,* was dichotomized using a median split at 3.6. There was not a significant interaction between *concern for physical appearance* and *value equity* on *satisfaction*, *F* (1, 505) = 33.09, *p* = 0.11. *Concern for physical appearance* did not moderate the link between *value equity* and *satisfaction* (see Table 4).

## 6. Discussion, Implications, and Conclusions

### 6.1. Discussion

This study investigated the impact of retail user experience with the perceived quality of AI VTOs on value equity and its subsequent effects on satisfaction and loyalty. First, as the retail user experience with perceived quality of AI VTOs, pragmatic quality, hedonic quality, and customization of AI virtual try-ons (VTOs) were found to be antecedents of value equity for AI VTOs (H1, H2, and H5), which in turn affected satisfaction with AI VTOs (H6). The findings of this study regarding the influence of pragmatic quality on cognitive and affective responses are in line with previous research regarding the effect of pragmatic attributes, like pragmatic realism and perceived usefulness, on behavioral outcomes in the VTO technology context (Ivanov et al., 2023; Lavoye et al., 2023; H. Yang & Kim, 2024). The finding of this study regarding the influence of hedonic quality on cognitive and affective responses is in line with previous research on AR-based shopping contexts (Kazmi et al., 2021; Poushneh & Vasquez-Parraga, 2017). The results of this study on the effect of customization on cognitive and affective responses are in agreement with previous research regarding the effect of customization features on cognitive and affective responses and behavioral outcomes in AR-based shopping (Q. Jiang et al., 2022; Kowalczuk et al., 2021) and in the virtual reality and VTO apps (Lau & Ki, 2021; Tawira & Ivanov, 2023). Thus, AI VTOs may enhance the retail user experience by featuring pragmatic quality, hedonic quality, and customization, resulting in a favorable value equity, which in turn increases user satisfaction with AI VTOs.

Second, this study found that retail users who are satisfied with AI VTOs were likely to exhibit repurchase loyalty using AI VTOs and brand loyalty (H7a & H7b), in line with the previous research in fashion retailing that demonstrated the significant influence of customer satisfaction on patronage behavior and retailer loyalty (M. J. Kim & Mullis, 2010). Third, this study confirmed the moderating effect of clothing in relation to self as structure on the positive relationship between value equity and satisfaction (H8). Clothing and self-alignment enhanced value outcomes and positively impacted satisfaction. The relationship between value equity and satisfaction was stronger for users whose clothing strongly aligns with their self-image. Conversely, this relationship was weaker for users who had a low level of clothing in relation to their self-image.

Fourth, contrary to our hypothesis, we found that the aesthetic quality and interactivity of AI VTOs were not determinants of value equity for AI VTOs (H3 & H4). This finding suggests a distinction between resonance experience and cognitive value evaluation in the context of AI VTO. While value equity indicates a cognitive judgment of the system, aesthetic quality reflects a combination of cognitive and affective responses. Moreover, as technology evolves, interactivity may be perceived as a baseline expectation rather than a differentiating attribute. Since the AI VTO stimulus used in this study may have lacked aesthetically pleasing and enjoyable attributes and advanced interactivity features, our participants might have been unable to recognize the importance and potential benefits of aesthetic quality and interactivity in AI VTOs. The limited interactive features of the stimulus may have constrained participants’ ability to fully experience interactivity. Furthermore, because AI VTOs tend to be goal-oriented tools, our task-oriented participants are likely to use the AI VTO tools to solve specific problems rather than to seek aesthetic appeal and high interactive elements of AI VTOs. Thus, our participants may prioritize functionality and utility (e.g., fit accuracy, realism, efficient try-on process) over aesthetics browsing and high interactivity when using AI VTO technology.

Fifth, this study found that concern for physical appearance did not moderate the link between value equity and satisfaction with AI VTOs (H9). It is possible that current AI VTOs may not yet offer the precision and realism necessary to address concerns regarding users’ diverse physical appearances thoroughly. Therefore, our participants with physical appearance concerns may have been unable to perceive the AI VTOs as a reliable tool to reduce their specific appearance concerns and anxiety about fit and look.

### 6.2. Theoretical Implications

This study applied the *Means–End Chain* model (Gutman, 1982) to the *quality–value–satisfaction–loyalty* chain (Gallarza et al., 2013) in the context of AI VTO. The theoretical implications of this study are threefold. First, this study validated the applicability of the *Means–End Chain model* in the AI VTO context. In this study, retail user experience was examined as a *means* state: (1) pragmatic quality, (2) hedonic quality, (3) aesthetic quality, (4) interactivity, and (5) customization. Value equity for AI VTO was examined as achieving the desired *end* state. Empirical evidence from our study confirmed that retail user experience with perceived pragmatic quality, hedonic quality, and customization of AI VTOs acted as the key drivers of value equity for AI VTOs in a *Means–End Chain model.* The pragmatic quality of being practical, reliable, informative, and useful reinforces the functional value of AI VTOs. The particularly strong contribution of hedonic quality in strengthening value perceptions contributes to the theory that experiencing emotional and rational stimulation is not merely supplementary but integral and essential to value formation in the AI VTO setting. Customization, a distinctive feature of AI-driven systems, extends the theory by highlighting adaptable personalization as another important quality through which value perception is formed and strengthening the *quality–value–satisfaction–loyalty* chain (Gallarza et al., 2013) in the digital retail environment. As a primary *means* for users, retail user experience with these three attributes of AI VTOs enabled the fulfillment of customer values for AI VTOs as desirable *end* states. These findings are in line with prior studies demonstrating the positive effects of the perceived utilitarian value and personalization of AI try-on technology (Gao & Liang, 2025), as well as the positive impact of perceived enjoyment of VTOs (Chen et al., 2024; H. Yang & Kim, 2024) on favorable behavioral outcomes. Surprisingly, the insignificance of both aesthetic quality and *interactivity* indicates a theoretical shift. In the AI-mediated shopping journey, our participants tended to prioritize practicality, reliability, informativeness, usefulness (pragmatic quality), emotional and rational stimulation (hedonic quality), and individualized tailoring (customization) over visual appeal and pleasurable experiences (aesthetic quality) and interactivity as means states. By integrating multiple AI-driven functionalities in VTO systems into a framework, this study demonstrates that AI VTO features can lead to desired value outcomes and that value formation in digital retail experiences is multidimensional in nature.

Second, this study enhanced the theoretical foundations regarding the *quality–value–satisfaction–loyalty chain* (Gallarza et al., 2013) by extending the traditional *quality–value–satisfaction* framework (Wang et al., 2004; Zeithaml, 1988) into the AI VTO context. This study confirmed that retail user experience with higher quality of AI VTO leads to greater perceived value equity of AI VTO and increased satisfaction with AI VTO, which in turn results in increased loyalty (i.e., repurchase loyalty and brand loyalty). The path from retail user experience with perceived quality to brand and repurchase loyalty was mediated through value equity for and satisfaction with AI VTO in the AI-mediated shopping journey. By situating AI VTO within this framework, this study demonstrates that technology-mediated retail experiences can be understood as a hierarchical consumer process.

Third, this study shows that the moderating role of clothing in relation to the self as structure acted as a boundary condition in the relationship between value equity and satisfaction. In other words, personal identity strengthens the link between value and satisfaction chain. This suggests that the impact of value equity on satisfaction may vary depending on how well the AI VTO experience aligns with the user’s identity. Our findings align with those of M. J. Kim and Mullis (2010) regarding the positive effect of self-congruity on satisfaction with retailers and retail loyalty. Thus, our findings further extend the theoretical framework and understanding of identity-related mechanisms in AI-based retail environments. Therefore, our study provided theoretical insights and an extension in the AI-mediated shopping journey by the integration of the *Means–End Chain model* (Gutman, 1982) into the *quality–value–satisfaction–loyalty* chain (Gallarza et al., 2013) framework.

### 6.3. Implications for Practice

In terms of retail user experience with perceived quality of AI VTOs, pragmatic quality, hedonic quality, and customization of AI VTOs were found to be antecedents of value equity for AI VTOs, which in turn affected satisfaction with AI VTOs. As to the managerial implications for practice, first, this study suggests that to ensure and enhance pragmatic quality, service providers need to optimize accuracy (i.e., precise avatar creation and fit accuracy) and realism in virtual try-on, fit and size recommendations, practical usability and utility, and the efficiency of the purchase flow. For example, an AI fashion app, Doji, provides features for users such as the ability to develop a customized avatar based on selfies and full-length body images. The app also allows for the user to try on an expanded assortment of clothing options by importing clothing URLs from online retailers. The app also provides a spontaneous navigation experience, shoppable links, and notifications. This simplifies the process from virtual try-ons to purchase and incorporates improvements in speed and integration of fit assessment tools (Bradley & Stokes, 2025).

Second, this study provides some suggestions to enhance hedonic quality for AI VTOs. Service providers could incorporate augmented reality and virtual reality technologies to create the customer’s immersive experience. Also, service providers could offer realistic visuals, mix-and-match outfits in a game-like environment, the option to save looks, and offer social sharing features to encourage users’ engagement and enhance their enjoyable experience.

Third, to improve customization for AI VTOs, service providers could incorporate AI-powered personalized recommendations, styling, immersive try-ons, and swappable layers, tailored to users’ preferences and past purchases and transactions. For instance, Glance, the advanced AI-powered platform, enables users to select and view outfits on AI-generated models acting as AI twins (Verma, 2025). It replicates fabric textures under various lighting conditions, provides outfit builders and style recommendations, and ultimately allows for users to create personalized digital closets (Verma, 2025).

Fourth, our study confirmed the moderating effect of clothing in relation to the self as structure. Service providers need to establish a strong connection between clothing and users’ identities within AI VTO systems. As identity-based marketing, our study suggested that retailers and facilitators need to allow for users to adjust VTO options such as fit, colors, or diverse styling with coordinated fashion items and accessories, environment/context-based overlays, body shape, and skin colors, to better align with users’ self-identity, self-perception, and self-expression. For example, well-known fashion brand Levi Strauss & Co. partnered with Lalaland.ai and created realistic AI models that reflect various body types, skin tones, and age groups for denim modeling (McQueen, 2023). Lalaland.ai’s technology enables the user to develop a customized AI avatar in minutes and incorporates specific measurements, skin tone, and even facial expressions (McQueen, 2023). Verma (2025) provides another example with the explanation of the “AI twin.” This is a customized user avatar that can be adjusted for body, style, and habits. Developing their own personal avatar comprises a digital self-practice that engages with the user and provides recommendations for clothing that align with the changes they make to their avatar to reflect their own identity. The close resemblance of the AI twin and the user generates emotional resonance and increases the personal relevance of the experience (Verma, 2025).

### 6.4. Conclusions

Our study contributes to the consumer behavior literature by demonstrating the impact of retail user experience with the perceived quality of AI VTOs on value equity and its subsequent effects on satisfaction and loyalty, as well as the moderating effect of clothing in relation to self as structure. The empirical analysis confirmed that pragmatic quality, hedonic quality, and customization of AI VTOs positively affected value equity for AI VTOs. However, aesthetic quality and interactivity of AI VTOs did not affect value equity for AI VTOs. The analysis found that value equity had a positive effect on satisfaction with AI VTOs, which consequently had a positive impact on repurchase loyalty using AI VTOs and brand loyalty. The findings also revealed that the effect of value equity for AI VTOs on satisfaction was stronger among users with high levels of clothing in relation to the self as structure than among those with low levels. However, concern for physical appearance did not moderate the link between value equity for and satisfaction with AI VTOs.

As to the theoretical implications, this study confirmed the applicability of the *Means–End Chain model* to the *quality–value–satisfaction–loyalty* chain in the AI VTO context. Our findings enhanced the theoretical foundations regarding the impact of quality on value as a *Means–End Chain model* in AI VTOs. In terms of managerial implications for practice, to ensure the pragmatic quality in AI VTOs, service providers need to optimize technical accuracy, virtual realism, practical usability, and transactional efficiency. To enhance the hedonic quality of AI VTOs, providers can incorporate immersive AR/VR technologies, mix-and-match environments, and social sharing features. Customization can be enhanced through AI-powered personalized styling and swappable layers tailored to an individual’s specific purchase history and preferences. Based on the moderating effect of clothing in relation to the self as structure in our proposed model, to ensure the virtual experience aligns with a user’s self-identity and expression, providers should offer diverse adjustment options, such as skin tone, body shape, and contextual overlays. This study helps identify which features of AI VTO systems most significantly affect cognitive and affective assessment and behavioral outcomes. Our implications can be applied to build a competitive market presence in AI VTOs and rapidly adapt to shifting customer demands in digital commerce.

### 6.5. Limitations and Future Research

This study has some limitations. First, this study relies on a non-probability, quota-based convenience sample from a pre-existing panel, so these findings may not fully generalize to the entire population of U.S. online apparel shoppers. Also, participants in this study were U.S. consumers. It would be worthwhile to explore further how our findings may generalize to additional global consumer groups in Europe and Asia, etc. Second, we used the Google AI VTO, which is in the early stages of development and implementation of AI VTOs. Every year, innovative AI VTO technologies evolve rapidly; therefore, future studies could use more advanced AI VTOs featuring personalized, diverse body types and appearances with fast transactions as a stimulus. Advanced attributes of AI VTOs may differently affect the retail user experience and their cognitive assessment, affective responses, and behavioral outcomes. Third, this study examined a cognitive evaluation pathway centered on value equity, which may underrepresent affective mechanisms. Future research could consider a sequential mediation analysis and alternative pathways, such as the direct effect of aesthetic quality on satisfaction. Fourth, in terms of the product category, our study focuses solely on apparel for AI VTOs. Our findings may not be generalizable to other sectors like cosmetics, home furnishing/furniture, or eyewear. Future studies could explore diverse product categories and then compare the results of the conceptual model across these different contexts.

## Figures and Tables

**Table 1:** Measurement model properties.

Measurement Items	CFA Standardized Factor Loading	Construct Reliability ^a^	AVE ^b^
Pragmatic Quality ** Using the AI VTO is: Pragmatic Quality-Practical		0.96	0.59
PQPR1: Confusing-clearly structured	0.77		
PQPR2: Impractical-practical	0.76		
PQPR3: Complicated-simple	0.78		
PQPR4: Difficult to learn-easy to learn	0.77		
PQPR5: Effortful-effortless	0.78		
Pragmatic Quality-Reliable			
PQRE1: Unpredictable-predictable	0.74		
PQRE2: Cumbersome-straightforward	0.79		
PQRE3: Unprofessional-professional	0.77		
PQRE4: Insecure-secure	0.77		
PQRE5: Irrelevant-relevant	0.80		
PQRE6: Unreliable-reliable	0.80		
Pragmatic Quality-Informative			
PQIN1: Too little information-too much information	0.67		
PQIN2: Shady-trustworthy	0.77		
PQIN3: Not personalized-personalized	0.78		
PQIN4: Highly decreases one’s awareness-highly increases one’s awareness	0.76		
Pragmatic Quality-Useful			
PQUS1: Highly decrease this online retailer’s service capabilities for customer shopping experience-highly augments one’s service capabilities for customer shopping experience	0.78		
PQUS2: Risky to use-safe to use	0.78		
PQUS3: Useless-Useful	0.78		
Hedonic Quality ** Using the AI VTO is: Hedonic Quality by Identification		0.95	0.56
HQID1: Alienating-integrating	0.77		
HQID2: Cheap-expensive	0.65		
HQID3: Tacky-stylish	0.76		
HQID4: Isolating-connective	0.77		
HQID5: Decreases one’s self image-improves one’s self-image	0.78		
Hedonic Quality by Emotional Stimulation			
HQES1: Repelling-appealing	0.78		
HQES2: Discouraging-motivating	0.78		
HQES3: Not absorbed-over absorbed	0.65		
HQES4: Not immersive-immersive	0.74		
Hedonic Quality by Rational Stimulation			
HQRS1: Ordinary-novel	0.75		
HQRS2: Dull-captivating	0.79		
HQRS3: Conservative-innovative	0.74		
HQRS4: Cautious-bold	0.76		
HQRS5: Unimaginative-creative	0.77		
HQRS6: Conventional-inventive	0.75		
Aesthetic Quality **		0.91	0.62
Using the AI VTO is: Aesthetic Quality-Cognitive AQCG1: Unattractive-attractive	0.75		
AQCG2: Aesthetically unpleasing-aesthetically pleasing	0.78		
AQCG3: Static-vivid	0.79		
AQCG4: Artificial-realistic	0.76		
Aesthetic Quality-Affective			
AQAF1: Unfriendly-friendly	0.79		
AQAF2: Annoying-enjoyable	0.80		
AQAF3: Unpleasant-pleasant	0.81		
Interactivity *		0.85	0.59
INT1: The AI VTO would enable me to see the merchandise from different angles.	0.75		
INT2: The AI VTO has tools that make product comparisons easy.	0.77		
INT3: Using the AI VTO is very engaging.	0.79		
INT4: Using the AI VTO is very dynamic.	0.74		
Customization *		0.86	0.61
CUST1: The AI VTO would enable me to purchase products that are suitable for me.	0.79		
CUST2: The AI VTO is perfectly suitable as per my shopping requirement.	0.78		
CUST3: The AI VTO would make me feel like a unique customer.	0.78		
CUST4: I am confident that the AI VTO would be customized as per my requirement.	0.78		
Value Equity **		0.88	0.66
VAL1: How would you rate your overall shopping experience through the AI VTO? (Extremely poor value to Extremely good value)	0.082		
VAL2: How would you rate the quality–price ratio of the AI VTO services provided by this brand? (Poor to Good)	0.80		
VAL3: How reasonable was the time you spent shopping through the AI VTO? (Highly unreasonable to Highly reasonable)	0.83		
VAL4: How worthwhile was the effort you put into the shopping experience through the AI VTO? (Not at all worthwhile to Very worthwhile)	0.80		
Satisfaction *		0.88	0.62
SAT1: I think that I made the correct decision to use this AI VTO.	0.79		
SAT2: The experience that I have had with this AI VTO has been satisfactory.	0.78		
SAT3: In general, I am satisfied with this AI VTO.	0.78		
Repurchase Loyalty *		0.86	0.60
RL1: I will use the AI VTOs the next time I buy apparel.	0.77		
RL2: I intend to shop again using the AI VTO provided by this brand.	0.78		
RL3: I probably won’t switch to another brand.	0.76		
RL4: I would recommend shopping using the AI VTO to my friends.	0.79		
Brand Loyalty * Think about a brand that offers the AI VTO.		0.80	0.58
LOY1: I consider myself to be loyal to this brand’s offline and online stores.	0.75		
LOY2: This brand’s website would be my first choice among similar retailers.	0.77		
LOY3: I will stop on another brand’s website as long as I can also access this brand’s offline and online stores.	0.76		

^a^ Composite Reliability = (∑ standardized loading)^2^/(∑ standardized loading)^2^ + ∑ measurement error; ^b^ Variance Extracted = ∑ (standardized loading)^2^/∑ (standardized loading)^2^ + ∑ measurement error; * Anchored with 5-point Likert-scale descriptors, from 1 = “Strongly disagree” to 5 = “Strongly agree; ** Anchored with a bipolar semantic differential 7-point.

**Table 2:** Discriminant validity of measures used in measurement and structural model.

	1	2	3	4	5	6	7	8	9
1. Pragmatic Quality	***0.59***								
2. Hedonic Quality	0.38	***0.56***							
3. Aesthetic Quality	0.43	0.41	***0.62***						
4. Interactivity	0.33	0.32	0.32	***0.59***					
5. Customization	0.33	0.32	0.22	0.32	***0.61***				
6. Value Equity of AI VTO	0.46	0.34	0.24	0.34	0.38	***0.66***			
7. Satisfaction with AI VTO	0.35	0.36	0.35	0.37	0.40	0.44	***0.62***		
8. Repurchase Loyalty	0.32	0.33	0.33	0.35	0.38	0.42	0.34	***0.60***	
9. Brand Loyalty	0.33	0.34	0.32	0.40	0.43	0.42	0.34	0.35	***0.58***

Note. The AVEs are on the diagonal, and the *R*^2^ (shared variance) is below the diagonal.

**Table 3:** Results: structural model.

Endogenous Constructs		Standardized Coefficient	t-Value ^a^
Value Equity for AI VTOs (R^2^ = 0.88)			
H1	Pragmatic Quality: Practicality, Reliability, & Information	0.28	2.90 **
H2	Hedonic Quality by Emotional and Rational Stimulations, & Identification	0.38	4.73 ***
H3	Aesthetic Quality: Cognitive & Affective	−0.03	−0.23
H4	Interactivity	−0.27	−1.69
H5	Customization	0.63	3.93 ***
Satisfaction with AI VTOs (R^2^ = 0.81)			
H6	Value Equity for AI VTOs	0.90	25.64 ***
Repurchase Loyalty (R^2^ = 0.97)			
H7a	Satisfaction with AI VTOs	0.98	27.82 ***
Brand Loyalty (R^2^ = 0.83)			
H7b	Satisfaction with AI VTOs	0.91	23.53 ***
Fit Statistics			
N		509	
χ^2^ (*df*)		3875.04 (1785)	
CFI		0.95	
NNFI		0.95	
IFI		0.95	
RMSEA		0.048	
SRMR		0.034	

^a^ SE, standardized estimate. ** *p* < 0.01, *** *p* < 0.001.

**Table 4:** Results: The moderating effect of clothing in relation to the self as a structure (H8) and physical concern (H9) on the relationship between value equity and satisfaction.

Hypotheses	*Levels*	*Estimate (Unstandardized Coefficient)*	Δ*χ*^2^	Δ*df*	Sig. *p*	Model Invariance	Moderating Effect
H8	Low	0.52	4.98	1	0.026	No	Yes
	High	0.61					
H9	Low	0.52	2.28	1	0.131	Yes	No
	High	0.58					

## Data Availability

The datasets generated in the current study are not publicly available due to privacy and ethical restrictions associated with the Institutional Review Board-approved research protocol and the participant consent process.
